# The effect of interictal epileptiform discharges on cognitive and academic performance in children with idiopathic epilepsy

**DOI:** 10.1186/s12883-020-01807-z

**Published:** 2020-06-06

**Authors:** Dazhi Cheng, Xiuxian Yan, Keming Xu, Xinlin Zhou, Qian Chen

**Affiliations:** 1 Department of Pediatric Neurology, Capital Institute of Pediatrics, Beijing, 100020 China; 2 National Key Laboratory of Cognitive Neuroscience and Learning, Institute of Cognitive Neuroscience and Learning, Beijing Normal University, Beijing, 100875 China

**Keywords:** Interictal epileptiform discharges, Cognitive function, Academic performance, Idiopathic epilepsy, Neuropsychological tests

## Abstract

**Background:**

Interictal epileptiform discharges (IEDs) have been proven to impair cognitive function. However, it is not clear whether IEDs disrupt academic performance in children with idiopathic epilepsy, and the contribution of cognitive function deficits to impaired academic performance has not been clarified. This study aimed to examine the cognitive deficits and academic impairment in childhood idiopathic epilepsy with IEDs.

**Methods:**

Ninety-seven childhood idiopathic epilepsy with IEDs, 77 childhood idiopathic epilepsy without IEDs, and 71 healthy controls completed a series of cognitive tests. We analyzed the cognitive performance in several domains including language, mathematics, psychomotor speed, spatial ability, memory, general intelligence, attention and executive functioning. Analysis of variance was conducted to compare the performance on all tests between the three groups.

**Results:**

Childhood idiopathic epilepsy with IEDs exhibited not only general cognitive deficits in processing speed, spatial ability, and attention, but also arithmetic impairment. Furthermore, general cognitive deficits could account for the impaired arithmetic performance in childhood idiopathic epilepsy with IEDs.

**Conclusions:**

Our study suggested that IEDs in children with idiopathic epilepsy affected both cognitive function and academic performance, and that the cognitive deficits may be responsible for arithmetic performance impairment.

## Background

Approximately 1–5% of the population exhibits epileptiform discharges on electroencephalography (EEG) [1]. Interictal epileptiform discharges (IEDs), meaning spikes, polyspikes, sharp waves, or spike and slow-wave complexes without observed clinical seizures, are commonly observed in children with epilepsy. Neuropsychological evidence indicates that childhood epilepsy often has negative effects on cognitive function [2]. Childhood absence epilepsy (CAE) and benign childhood epilepsy with centrotemporal spikes (BECTS) are the most common forms of idiopathic epilepsy. Although both are frequently associated with a good prognosis involving remission of seizures and epileptic discharge before puberty, specific cognitive dysfunctions still persist [3–5].

Epilepsy syndromes manifesting with IEDs are detrimental to cognitive function. Recently, two studies found that frequent IEDs can impair cognitive performance in children [6] and adult patients [7]. However, it is unclear whether IEDs are associated with disrupted academic performance in children with idiopathic epilepsy, and the relationship between general cognitive ability and academic performance in those patients has not been clarified. In this study, we examined the cognitive abilities and academic performance of children with idiopathic epilepsy with or without IEDs to test the hypothesis that childhood epilepsy patients with IEDs would exhibit specific cognitive deficits and academic impairment compared to controls.

## Methods

### Participants

Children were diagnosed with epileptic syndromes according to the International League Against Epilepsy criteria [8–10]. For inclusion in the study, children with epilepsy had to have experienced at least one seizure in the past year with a history of at least one certain seizure and the occurrence of clear epileptiform activity on EEG or at least two certain seizures (> 24 h apart).

The participants presented with one of three childhood idiopathic epilepsy: CAE, BECTS, or febrile seizure plus (FS +). The diagnosis of CAE was based on clinical evidence of typical absence seizures, especially induced by hyperventilation, and EEG results showing the typical 3–4 Hz generalized spike-and-wave complexes. BECTS patients were excluded if they had abnormal magnetic resonance imaging (MRI) results and a non-rapid eye movement (NREM) sleep discharge index ≥50% [7]. The patients with FS + were selected according to the International League Against Epilepsy criteria.

Children were excluded if they had ESES (i.e., electrical status epilepticus during slow-wave sleep), defined by a discharge index > 85% during a slow-wave sleep period. Patients with prior epileptic surgery and etiologies of brain structure abnormalities, such as cortical dysplasia or acquired lesions in MRI, were excluded. Previous studies showed that epilepsy patients with an NREM sleep discharge index > 50% frequently had cognitive impairment. Although clinically majority of the patients have an NREM sleep discharge index < 50%, there is still no large sample study to prove whether these patients have cognitive impairment. We excluded patients without an acceptable sleep and awake video-EEG and those with an NREM sleep discharge index > 50%. Patients unable to complete neuropsychological tasks independently were also excluded.

Participants were recruited from the Department of Pediatric Neurology at the Capital Institute of Pediatrics. The study sample included 93 children with BECTS, 33 children with CAE, and 48 children with FS + (89 boys and 85 girls, age range: 6–15 years). The patients were divided into two groups (childhood epilepsy with IEDs and childhood epilepsy without IEDs) according to the EEG monitoring. There were no significant differences between the two groups in terms of age or gender (Table 1). In addition, the control group included 71 healthy participants (35 boys and 36 girls) who were recruited from a primary school and a junior high school in Beijing. The mean age at the time of testing was 10.1 years (range: 6–15 years). The control group was matched with the epilepsy group for age and gender. Approval for this project was granted by the Human Research Ethics Committees at the Capital Institute of Pediatrics (approval no.: SHERLL 2015023). Written informed consent was obtained from the parents or guardians of all the patients.

**Table 1:** Characteristics of childhood epilepsy with and without IEDs

Characteristics	Childhood epilepsy with IEDs	Childhood epilepsy without IEDs	Controls	*p*-value
Number of participants	97	77	71	
Age (y), mean ± SD (range)	10.3 ± 2.1 (6–15)	10.8 ± 2.0 (6–15)	10.1 ± 2.5 (6–15)	0.12
Males/females	51/46	38/39	35/36	0.97
Socioeconomic status				
Urban	44.3%	50.6%	56.3%	0.30
Rural	55.7%	49.4%	43.7%	
Epilepsy classification				0.07
BECTS	60.8%	44.2%	N/A	
CAE	14.4%	24.7%	N/A	
FS +	24.7%	31.2%	N/A	
Antiepileptic drugs				
Yes	78.4%	83.1%	N/A	0.43
No	21.6%	16.9%	N/A	
Seizure in EEG monitoring				
Yes	36.1%	7.8%	N/A	0.001
No	63.9%	92.2%	N/A	

IEDs: interictal epileptiform discharges; SD: standard deviation; BECTS: benign epilepsy with centrotemporal spikes; CAE: childhood absence epilepsy; FS +: febrile seizure plus; N/A: not applicable

### EEG monitoring

All the children were monitored using video-EEG for 4 h. Sleep deprivation was performed before EEG monitoring. The EEG waveforms during sleep and waking time were recorded. Recording electrodes were placed according to the 10–20 system. EEG was performed by experienced technicians in a quiet and dark environment. EEG monitoring recorded the spike and wave discharges during the ictal period, or an absence attack induced by hyperventilation. EEG discharges, such as non-clinical seizures and especially non-convulsive seizures, were analyzed by pediatric epilepsy specialists. Epileptiform EEG discharges during episodes were analyzed with the indices of slow-wave sleep periods (i.e., the ratio between discharge time and total time in the period). IEDs were defined as spikes or spike-wave complexes, isolated or occurring serially (in runs) without evident clinical signs of a seizure. The patients were divided into two groups according to the presence or absence of IEDs in the EEG monitoring. For childhood idiopathic epilepsy without IED, there was no interictal epileptiform discharges in the 4-h video EEG after sleep deprivation within 72 h of the cognitive task.

### Neuropsychological tests

All the tests were programmed using web-based applications in the Online Experimental Psychological System. Neurocognitive tests included seven cognitive tasks [11]. The original tests materials of mental rotation and visual tracing were paper-based versions, which were adapted into computerized cognitive tests. For all but two tests, the children indicated their responses by pressing one of two keys (“P” or “Q”) on a computer keyboard; for visual tracing and the Wisconsin Card Sorting Test, they used the mouse to click on the correct answer. Table 2 provides details regarding the target cognitive domains and specific abilities assessed by each test. Word semantics and simple subtraction were used to assess academic performance. Participants’ responses were automatically recorded and sent via the Internet to a server. Participants’ EEG and cognitive test assessment were both performed within a 2-week period. For choice reaction time, mental rotation, simple subtraction, word semantics, Raven’s progressive matrices, visual tracing, and the Wisconsin Card Sorting Test, split-half reliability ranged from 0.83 to 0.93 according to previous studies [12, 13].

**Table 2:** Neuropsychological tests battery

Domain	Ability	Tests
Mathematics	Arithmetic**/**computational fluency	Simple subtraction
Language	Semantic comprehension	Word semantics
Psychomotor speed	Processing speed	Choice reaction time
Spatial ability	Spatial perception	Mental rotation
General Intelligence	Non-verbal matrix reasoning	Raven’s Progressive Matrices
Attention	Visual attention	Visual tracing
Executive functioning	Response inhibition and mental flexibility	Wisconsin Card Sorting Test

#### Choice reaction time

This previously described task was used to evaluate processing speed [14]. In this test, a white dot was presented on a black screen, either to the left or right of a fixation cross in each trial. The position of the dot was within 15° of the visual angle from the fixation cross. Participants were asked to press the “P” or “Q” key if the dot appeared on the left or right of the fixation cross. There were 30 trials in total (15 trials each with the dot on the left and right). Inter-stimulus intervals varied randomly between 1500 ms and 3000 ms.

#### Mental rotation

This task was adapted from that used by Vandenberg and Kuse [15] to evaluate spatial perception. The task has been used in previous studies, with split-half reliabilities from 0.87 to 0.91. In each trial, there was one three-dimensional image presented on the upper part of the screen and two more presented on the lower part of the screen. The non-matching image was a rotated mirror image of the target. Participants identified which image on the lower part of the screen matched the image on the upper part of the screen using only mental rotation. Participants pressed the “P” or “Q” key to choose the right answer. This was a time-limited (3-min) test with total 180 trials. The rotation angles of the matching images ranged from 15° to 345° with an interval of 15°.

#### Simple subtraction

This time-limited (2-min) task evaluated mathematical ability. There were 92 simple-subtraction problems (e.g., 6 minus 2, 17 minus 8) with minuends of 18 or smaller and one-digit number differences [12]. Two candidate answers were presented beneath each problem, and participants chose the “P” or “Q” key to indicate the right answer. The incorrect candidate answer ranged from the correct answer minus 3 to the correct answer plus 3 (i.e., ±1, ±2, or ± 3).

#### Word semantics

This task was similar to the one used in a previous study to evaluate language ability [16]. The materials were selected from primary school textbooks (first to ninth grade). In each trial, a sentence was presented in the middle of the computer screen with a word missing. Participants needed to select one of two candidate words presented beneath the sentence by pressing the “P” or “Q” key. The stimulus was presented on the screen until the participant responded.

#### Raven’s progressive matrices

This task was a simplified version of Raven’s Progressive Matrices test to evaluate general intelligence [17]. Participants were asked to identify the missing segment of a figure’s pattern. Two candidate answers were presented side by side beneath each problem. Participants were asked to press the “P” or “Q” key to choose the correct answer. This was a time-limited (3-min) task consisting of 80 trials.

#### Visual tracing

This was a time-limited (4-min) task adapted from the Groffman’s visual tracing test [18] to evaluate visual attention. In each trial, several curved lines were interwoven within a square, starting from the left side and ending on the right side of the square. Participants were asked to track a specified line from the beginning to the end with only their eyes (i.e., they were not allowed to use the cursor, a finger, or an object to trace) and mark the correct end point. This task became more difficult as the total number of lines increased.

#### Wisconsin card sorting test

This task was adapted from the manual version of the Wisconsin Card Sorting Test [19]. It evaluated executive function and assessed response inhibition and mental flexibility. This was a time-limited (20-min) task involving stimulus and response cards. The participant was instructed to match the response card to one of the stimulus cards (according to one of three principles, color, shape, or quantity). There were four types of stimulus cards: (a) one red triangle, (b) two green stars, (c) three yellow crosses, and (d) four blue circles. The participants had to try to guess the matching principle on the basis of facial feedback (a smiling or crying face to indicate “correct” or “incorrect”) received on each response.

### Procedure

Each participant completed the computerized test battery, which was administered individually in two 45-min sessions in an examination room. All test procedures were presented on a computer screen, and instructions were given orally. For each test, instructions were given first, followed by a practice session. After the children finished the practice session and had no more questions, they could press the space key to begin the formal test. The tasks were administered in the same order for all participants. Each participant was monitored by one tester who was trained in the standardized testing procedures.

### Statistical analysis

For all tests except the choice reaction time test, corrected scores were calculated by subtracting the number of incorrect responses from the number of correct responses to control for the effect of guessing [20]. For the choice reaction time test, each participant’s median reaction time was calculated. Analysis of variance (ANOVA) was conducted to compare the performance on all tests between the three groups, with post hoc pairwise comparisons using the Bonferroni correction.

Analysis of covariance (ANCOVA) was conducted to examine the relationships among general cognitive processing, language processing, and mathematical abilities. The general cognitive measures were also treated as covariates to identify differences in academic performance between the groups. To analyze the effects of socioeconomic status and epilepsy-related clinical variables (epilepsy classification, antiepileptic drugs, and seizures) on cognitive test scores, simple regression analysis was used. All *p*-values for the main effects and interactions were corrected using the Greenhouse-Geisser method. In case of significant effects, pairwise comparisons of performance were conducted.

## Results

### Population characteristics

The demographic and clinical characteristics of the participants are summarized in Table 1. There were no significant differences between the groups of childhood epilepsy without IEDs, childhood epilepsy with IEDs, and controls in age (*F* = 2.17, *p* = 0.12), gender (χ^2^ = 0.07, *p* = 0.97) or socioeconomic status (χ^2^ = 2.40, *p* = 0.32). There were no significant group differences between childhood epilepsy with IEDs and without IEDs with respect to epilepsy classification (χ^2^ = 5.25, *p* = 0.07) or antiepileptic drugs (χ^2^ = 0.62, *p* = 0.43). There were, however, significant group differences in seizure (χ^2^ = 19.07, *p* < 0.001).

### Cognitive function

The results of the neurocognitive tests are presented in Table 3. The ANOVA showed that there were significant group differences in the three tests of general cognitive processing: *F* (2, 242) = 11.12, *p* < 0.001, η_p_^2^ = 0.084 for choice reaction time; *F* (2, 242) = 5.25, *p* < 0.01, η_p_^2^ = 0.042 for mental rotation; and *F* (2, 242) = 6.86, *p* < 0.01, η_p_^2^ = 0.054 for visual tracing. Pairwise comparisons showed that both groups of childhood epilepsy with and without IEDs had impaired performance compared with the control group (all *p*s < 0.05), but childhood epilepsy without IEDs did not differ significantly from the childhood epilepsy group with IEDs. For simple subtraction, there were significant group differences, *F* (2, 242) = 3.64, *p* < 0.01, η_p_^2^ = 0.054. Pairwise comparisons showed that only the group of childhood epilepsy with IEDs performed significantly worse than the control group (*p* < 0.05), but the group of childhood epilepsy without IEDs did not differ significantly from the other two groups (vs. childhood epilepsy with IEDs, *p* = 0.82; vs. control group, *p* = 0.38).

**Table 3:** Neurocognitive performance scores (mean and SD) and ANOVA for cognitive test results of childhood epilepsy with and without IEDs

Tasks	Childhood epilepsy with IEDs	Childhood epilepsy without IEDs	Controls	*F*-value
Choice reaction time	521.48 (132.33)***	511.74 (120.41)**	425.43 (163.84)	11.12***
Mental rotation	14.9 (10.43)*	14.65 (10.20)*	19.72 (11.94)	5.25**
Simple subtraction	33.53 (11.45)*	35.66 (12.83)	38.89 (14.21)	3.64*
Word semantics	20.95 (11.73)	21.96 (11.01)	22.99 (14.08)	0.57
Raven’s Progressive Matrices	15.78 (7.70)	16.09 (6.94)	17.58 (6.53)	1.40
Visual tracing	11.49 (5.94)*	11.34 (6.79)*	14.89 (7.36)	6.86**
Wisconsin Card Sorting Test	66.33 (16.30)	69.18 (18.81)	67.51 (15.57)	0.61

*p < 0.05; ** *p* < 0.01; *** *p* < 0.001

IEDs: interictal epileptiform discharges; SD: standard deviation, ANOVA: analysis of variance

* Differs from control subjects (*p* < 0.05)

** Differs from control subjects (*p* < 0.01)

*** Differs from control subjects (*p* < 0.001)

The ANCOVA indicated that after controlling for the three cognitive ability test scores (choice reaction time, mental rotation, and visual tracing), group differences for simple subtraction were not observed, *F* (2, 239) = 0.94, *p* = 0.39. In the linear regression analysis, the effect of socioeconomic status and clinical variables on cognitive test scores was analyzed. There was no significant effect in socioeconomic status, epilepsy classification, antiepileptic drugs, and seizures (all *p*-values > 0.05).

## Discussion

This study aimed to examine the cognitive deficits and academic impairment in childhood idiopathic epilepsy with IEDs. The results demonstrated that children with idiopathic epilepsy with and without IEDs showed general cognitive deficits in processing speed, spatial ability, and attention. Only the group of childhood epilepsy with IEDs showed academic impairment in arithmetic compared with healthy controls. There was no significant difference between childhood epilepsy with IEDs and childhood epilepsy without IEDs. We further analyzed the relationships between general cognitive deficits and impaired arithmetic performance and found that general cognitive deficits could account for the impairment in arithmetic performance. These results suggest that IEDs in childhood idiopathic epilepsy are associated with disrupted mathematical performance, which is related to general cognitive deficits.

The current investigation found that general cognitive deficits in processing speed, spatial ability, and attention occurred in childhood epilepsy with and without IEDs. These general cognitive deficits may be neurobehavioral comorbidities of idiopathic epilepsy. This result is consistent with other studies concerning general cognitive dysfunctions in epilepsy. First, several studies indicated that IEDs in patients with epilepsy had a disruptive effect on information processing speed [6, 21], with even a low percentage of IEDs (1%) impairing information processing speed in childhood epilepsy. Second, most patients in the present study were children with BECTS. Previous studies suggested that BECTS exhibits a brain activity abnormality in the middle frontal gyrus and superior parietal gyrus, areas related to spatial ability [22, 23]. Therefore, both groups of children with epilepsy, with and without IEDs, showed a cognitive deficit in spatial ability. Third, children with centrencephalic epilepsy were shown to have attention deficits, without recording actual subclinical seizures and EEG discharges [24]. In a prospective open-label study, children with epilepsy showed frequent IEDs and attention and/or learning difficulties [25].

Furthermore, our results indicate that only the group with IEDs showed academic impairment in arithmetic. In children with BECTS examined by EEG-fMRI, the local interictal discharges could impair larger networks, as the frontal brain areas were functionally disturbed during the occurrence of Rolandic spikes [26]. Children with CAE may have disrupted long-term psychosocial outcomes, as it is possible that IEDs progressively disrupt thalamo-frontal neuronal networks, leading to deficits in attention functions [27]. FS + patients showed visual–motor impairments which affected arithmetic performance [11, 28]. Moreover, arithmetic processing is associated with the medial frontal lobe [29]. Therefore, our finding that childhood epilepsy with IEDs only had cognitive deficits in mathematical academic achievement is consistent with previous findings. However, there was no significant difference between childhood idiopathic epilepsy with IEDs and childhood idiopathic epilepsy without IEDs regarding cognitive abilities and academic achievement. The IEDs might not be the unique influencing factor to the cognitive function of idiopathic epilepsy. Some other epilepsy-related clinical variables (e.g., epilepsy classification, antiepileptic drugs, and seizures) also affected the cognitive abilities. It is possible that these influencing factors interaction with each other, together contributed to the cognitive deficits in children with idiopathic epilepsy. Thus, childhood idiopathic epilepsy with IEDs did not differ from childhood idiopathic epilepsy without IEDs in cognitive abilities and academic achievement.

In the present study, general cognitive deficits including processing speed, spatial ability, and attention could account for arithmetic impairment in epilepsy patients. Consistent with the present findings, the most prominent effects of IEDs are attention and motor speed [30]. Psychomotor speed and spatial ability could account for a significant amount of additional variance when predicting mathematical ability [31]. In addition, attention is significantly associated with arithmetic performance; for example, 42% of children with dyscalculia also experience attention deficits (30). Moreover, visual attention ability can predict mathematical but not reading performance (31). To sum up, childhood idiopathic epilepsy with IEDs showed not only general cognitive deficits but also arithmetic performance deficits. General cognitive deficits may be responsible for arithmetic performance deficits in childhood idiopathic epilepsy with IEDs.

This study has some limitations, namely the statistical approach and incomplete assessment for mathematical ability. ANCOVA was used to examine the relationships between cognitive abilities and arithmetic performance. It may be possible to use structural equation modeling [32] to explain the interaction effect between general cognitive abilities and academic achievement. By using a typical assessment approach of arithmetic fluency as a measure of arithmetic performance [33], we found that impaired arithmetic performance occurs in childhood epilepsy with IEDs, and that related general cognitive deficits contribute to arithmetic performance in these patients. This implies that interventions in childhood epilepsy with IEDs should focus on both general cognitive deficits and academic underachievement. In addition, participants’ EEG monitoring and cognitive test assessment were both performed within a two-week period. Thus, it demonstrated the transient effect of IEDs on cognition rather than long-term cognitive impact.

## Conclusion

Our findings indicated that childhood idiopathic epilepsy with IEDs is associated with arithmetic performance deficits. This study also illustrated that general cognitive deficits accounted for impairment in arithmetic performance. We conclude that IEDs in children with idiopathic epilepsy affected both cognitive function and academic performance, especially arithmetic ability. The cognitive deficits may be responsible for arithmetic performance impairment. Future follow-up studies are required to investigate the further impact of IEDs on long-term cognitive outcomes.

## Data Availability

All data generated or analyzed during this study are included in this published article.

## Abbreviations

IEDs: Interictal epileptiform discharges
CAE: Childhood absence epilepsy
BECTS: Benign childhood epilepsy with centrotemporal spike
FS+: Febrile seizure plus
MRI: Magnetic resonance imaging
NREM: Non-rapid eye movement
EEG: Electroencephalography
ANOVA: Analysis of variance
ANCOVA: Analysis of covariance
SD: Standard deviation
